# The sleeping brain regulates to the edge of chaos

**DOI:** 10.1186/1471-2202-15-S1-O19

**Published:** 2014-07-21

**Authors:** Moira L Steyn-Ross, Alistair Steyn-Ross, Jamie W Sleigh

**Affiliations:** 1 School of Engineering, University of Waikato, Hamilton 3240, New Zealand; 2 Waikato Clinical School, University of Auckland, Hamilton 3240, New Zealand

## 

One of the most intriguing ideas in complexity theory is the notion that some systems can organize dynamically to a point critically poised between order and disorder, hovering at the so-called “edge of chaos”. It has been proposed that the computational performance of neural networks is optimized when close to the order–disorder phase transition. In this presentation we explore the novel hypothesis that the human brain may be operating at the edge of chaos during slow-wave sleep (SWS), the deepest phase of NREM (non-rapid-eye-movement) sleep.

**Figure 1:**
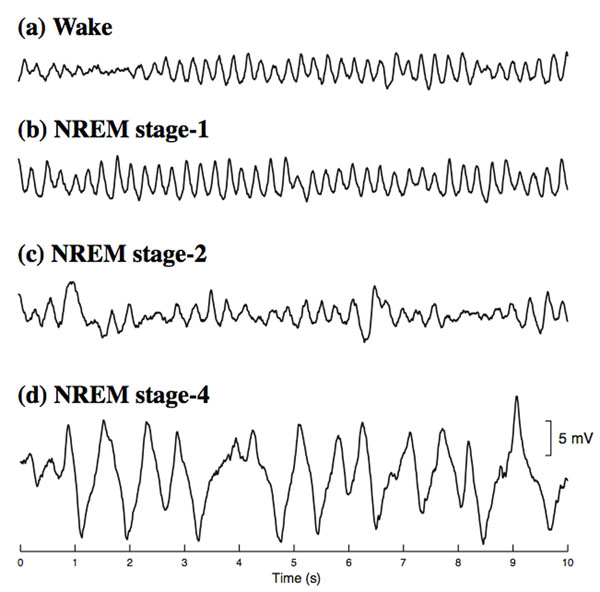
Model-generated sleep electrocorticograms for descent from wake to deep NREM. Model predictions are the excitatory soma voltage recorded at one point on a 120×120 cortical grid after filtering with a 0.5-Hz high-pass filter.

We build on an existing continuum model of the cortex [1] to incorporate known changes in specific neurotransmitter concentrations—GABA increase with simultaneous acetylcholine (ACh) decrease—during descent from wake into natural SWS [2]. The GABA boost is modeled as an anesthetic-like prolongation of the inhibitory postsyaptic potential (IPSP) paired with a restriction of gap-junction connectivity, while ACh suppression reduces resting cell voltage but enhances excitatory synaptic efficiency. Our model is able to produce a plausible sequence of time-series for EEG progression through the stages of NREM sleep (see Figure 1).

These sleep-induced neurotransmitter changes can have profound effects on cortical stability: alterations in inhibitory gap-junction connectivity controls a pattern-forming Turing instability, and manipulations of IPSP duration can lead to Hopf temporal oscillations which, in a pathological limit, can lead to whole-of-cortex seizure. We argue that normal brain function requires a balance between Turing and Hopf instabilities, and that descent into deep sleep entails a rebalancing in favor the Hopf instability. Model simulations predict that the spatiotemporal patterns for NREM sleep stages-1 to -4 are chaotic, showing exponential trajectory divergence from closely similar starting conditions. In contrast, the seizure state is highly ordered and non-chaotic. Since most sleepers do not proceed to seizure, we posit the existence of a protective mechanism that regulates the naturally sleeping brain so that it remains close to—but does not cross—the disorder/order boundary during deepest sleep.

There is clinical evidence that high cortical activity is associated with closure of gap-junctions [3]. This has motivated a learning rule that regulates the gap-junction conductivity based on the spatial covariance of inhibitory firing-rate activity across the two-dimensional cortical grid. We find that this rule enables the cortex to regulate its slow-wave dynamics from chaotic to marginally-ordered, and that regulation failure typically leads to seizure onset.
